# Peer Education and Peer Counselling for Health and Well-Being: A Review of Reviews

**DOI:** 10.3390/ijerph19106064

**Published:** 2022-05-17

**Authors:** Keith James Topping

**Affiliations:** School of Education, University of Dundee, Dundee DD1 4HN, UK; k.j.topping@dundee.ac.uk

**Keywords:** peer education, peer counselling, peer support, health, well-being, narrative reviews, systematic analyses, meta-analyses, effect, outcome

## Abstract

Peer education and peer counselling for health and wellbeing have been recognized as complementary approaches to professional intervention for over 50 years, but it is relatively recently that research into effects has become adequate. Potentially, they have advantages in reaching where professionals cannot, but it has not been clear if that potential is fulfilled, although the measurement of effects is difficult. The present paper examines 58 narrative and systematic reviews and meta-analyses on the topic. In peer education, there were many reviews of sexual health and of HIV/AIDS interventions, followed by reviews of various medical conditions and in the context of prisons. More general reviews covered a wider field. In peer counselling, there were several reviews of breast-feeding and mental health. Many early reviews complained of the lack of evaluation; then, later reviews found knowledge gains but not behavior gains; then, still later reviews found both knowledge and behavior gains. Thus, peer education and counselling appear effective but only if organizational factors are well managed and the cultural context of the country respected. The implications for future practice, policy and research were outlined.

## 1. Introduction

Peer education can be defined as “peers offering credible and reliable information about sensitive life issues and the opportunity to discuss this in an informal peer group setting” [1] (p. 7). Peer counselling can be defined as “people from similar groupings who are not professionals who help to clarify life problems and identify solutions by listening; clarifying; feeding back; summarizing; questioning and being positive, supportive and reassuring and then helping plan, organize and problem-solve”. [1] (p. 7). These definitions are quite elderly, but no more recent ones were identified in the literature. Peer education is usually in groups, while peer counselling may be one-to-one, in groups or a mixture of the two. However, these definitions are highly contestable, many authors make no attempt to define activities as one or the other and, indeed, peer education is sometimes very difficult to distinguish from peer counselling.

Arguably, peer education has more chance of permeating the peer group and changing behavior than information-giving by professionals. Although now used for a wide variety of challenging areas of personal, social and health education, peer education is not an easy option and is inherently difficult to quality control. This is also true of peer counselling, which is in a sense the more individualized remedial and palliative counterpart to the wider preventative aspirations of peer education. Evidence that peer counselling is as effective as counselling by professionals has been found in the literature for many decades (e.g., [2,3]). Neither peer education nor peer counselling is at all new, but an increasing number of interesting and well-designed studies are now available to help refine methods and improve effectiveness.

Peer education and peer counselling projects can be found in primary and secondary schools, in further and higher education, in work training and the workplace and in community settings. A very important feature of these methods is that they reach beyond formal teaching: they can bridge the traditional gap between the educational institution and the “real world”, reaching where professionals cannot be or cannot go. This does of course make them inherently difficult to quality control and evaluate. Many programs using these methods seek gains for the helpers as well as the helped; to be a helper may actually be more therapeutic than being helped. However, the helpers should be sociologically similar to those who are to be helped, or their credibility will be limited [4]. 

### Aim of This Paper

Many organizations around the world are using peer education and peer counseling in the hope of promoting healthy behaviors and well-being, sometimes as a means to compensate for lack of professional staff and sometimes in an effort to integrate better with the natural environment of those to be helped, and obviously, their efforts need to be informed by the research base. The historical perspective above indicates many years of research into these topics, but has that research improved in quality, to the extent that it now offers convincing evidence? This review of reviews seeks to appraise the field of peer education and counselling from the perspective of different published reviews over the years, which are of different styles: narrative, systematic or meta-analytic. It asks the question: What is the evidence that such programs are effective, and is the evidence more convincing in some areas of operation than others?

## 2. Methods

**Figure 1:**
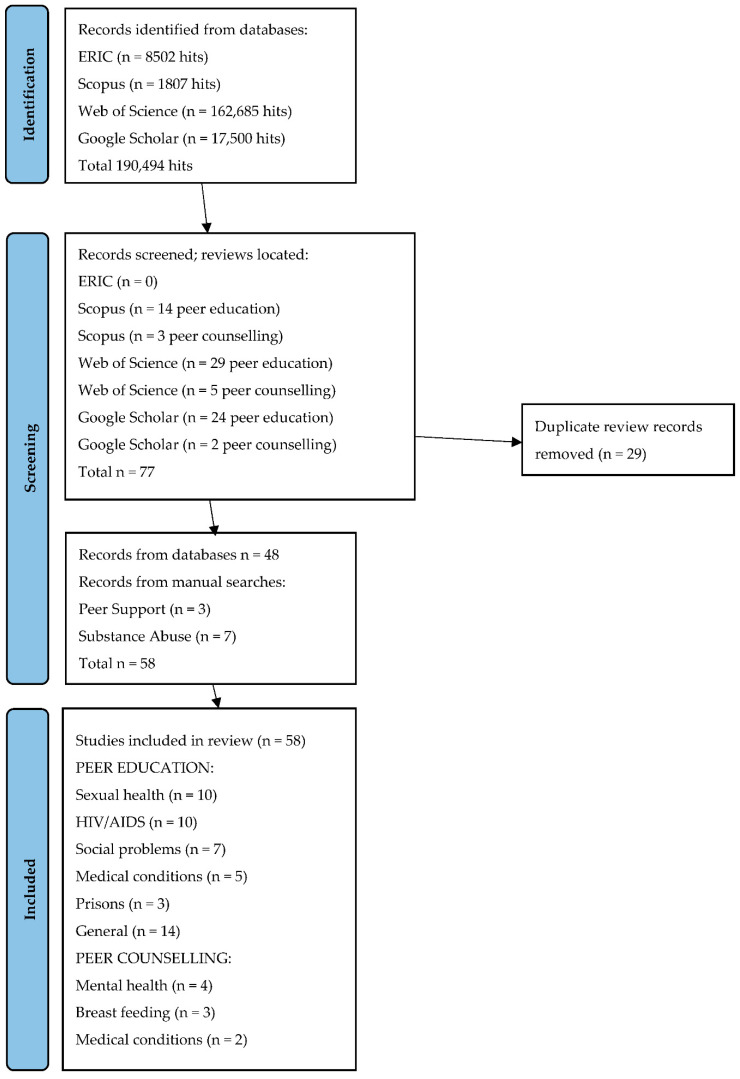
PRISMA Flowchart of Search Strategy.

This paper set out to be a systematic review of peer education and peer counselling for health and well-being. The keywords were “peer education” OR “peer counselling” OR “peer counseling” AND health OR well-being OR wellbeing. To ensure that all possible reviews were extracted, the search was then run again with “AND reviews” added to each of the search terms. Figure 1 outlines the search strategy. The databases searched were ERIC, Scopus, Web of Science and Google Scholar. ERIC yielded 8502 hits, Scopus 1807, Web of Science 162,685 and Google Scholar 17,500.

The criteria for inclusion were that the item concerned peer education or peer counselling (even if it did not use either term), was a peer-reviewed journal article (so books, chapters, doctoral theses, research reports and conference papers were excluded), was in the English language and reported quantitative and/or qualitative data evidencing the conclusions or was a review of such evidence. No year limits were set, so items from the beginnings of the field up to 2022 were retrieved.

Very different numbers of papers were indicated as relevant by their title and abstract from the different databases. 

ERIC: the search yielded only three single studies of peer education and six single studies of peer counselling. The search for reviews had no results.

Scopus: the search yielded 23 single studies of peer education and five single studies of peer counselling. The search for reviews yielded 14 reviews of peer education and three reviews of peer counselling. 

Web of Science: the search based on the initial search terms was ineffective. Accordingly, I entered “peer education” AND review and got 307 results. I then entered “peer counseling” OR “peer counselling” AND review and got 371 results. Duplicates were eliminated and these searches together yielded 29 reviews of peer education and five reviews of peer counselling. 

Google Scholar—the search for items yielded 44 single studies of peer education and 10 single studies of peer counselling. The search for reviews yielded 24 reviews of peer education and two reviews of peer counselling.

I then sought to eliminate duplicates across databases. This left 42 reviews of peer education and six reviews of peer counselling. 

The single studies were interesting, not least as latterly they included a number of randomized controlled trials, which indicated that the quality of research had greatly improved in recent years, although, of course, RCTs also have their problems. However, given the large volume of 48 reviews, I decided to disregard the single studies and focus on the reviews. These were supplemented by a further three reviews on “peer support” (a vague term which has similarities to peer counselling) [5,6,7], which were retrieved manually. It was surprising that there were no reviews on smoking, alcohol abuse, drug use or obesity. Consequently, I conducted a manual search under these terms, finding one review on smoking [8], two on drug use [9,10], one on alcohol abuse [11] and three on obesity [12,13,14]. Thus, 58 reviews were finally included in this paper.

Considering the reviews which were extracted, some were narrative, most professed to be systematic analyses and a few were meta-analyses. Some were of good quality and others less so, and although this showed some relationship to the age of the review, in other cases it seemed largely irrespective of this. Some reviews focused on young people, others on adults and some on a mixture of the two. Later reviews were more likely to include randomized controlled trials (RCTs) or be exclusive to RCTs. A minority of these reviews gave Effect Size (ES), mean standard deviation (MSD), odds ratios (OR) or risk ratios (RR) as a measure of overall impact, which was surprising given that a number of them stated they were following PRISMA guidelines (http://www.prisma-statement.org (accessed on 1 March 2022)). The peer education reviews fell into five categories: Sexual health (10 reviews), HIV/AIDS (10 reviews), Social problems (seven reviews), Medical conditions (five reviews), Prisons (three reviews) and General (14 reviews). The peer support reviews were merged with the peer counselling reviews. The peer counselling and support reviews fell into three categories: Mental health (four reviews), Breast-feeding (three reviews) and Medical conditions (two reviews).

## 3. Results: Peer Education

“More Education” is routinely prescribed as the panacea to cure all the social ills of contemporary society. However, traditional personal and social education by professionals such as teachers has proved singularly ineffective in ameliorating these ills. This was realized as long ago as the mid-1970s by authors such as Evans [15]. By the 1980s, the field had developed to the extent that it could be said that “peer education needs to be incorporated in nearly every facet of the health education curriculum” [16]. However, supposedly “psycho-social” interventions were still simplistic and individually oriented [17]. Any assumption of a homogeneous peer group or “teen culture” was very naïve: young people are not all the same. This might also be true of siblings as peers, but I will not discuss sibling helping in this paper. 

As early as 1983, a review was offered of secondary school peer education programs [18]. In 1985, there was a review of peer education in smoking prevention [19]. This was followed by two reviews of peer education in the prevention of alcohol problems [20,21]. Peer education is found in areas such as drug abuse, drinking and driving, contraception, sexually transmitted diseases, diet and nutrition, violence prevention, suicide prevention, gang membership, dealing with divorce and loss, drop-out prevention, coping with chronic/terminal illness, rape awareness, sexual harassment and post-traumatic stress syndrome [4]. Many of these topics were found as difficult to deal with by professional teachers. However, did the peer education affect the actual behavior of the participants in the longer term? This is of course another question and a key question in evaluation.

Peers can speak to each other directly in their own language, with the credibility of participants in the same culture who have perhaps had similar experiences, and without any overtones of social control and authoritarianism. Peers listen to each other. Of course, they might not have their facts exactly right, and training seeks to give some reliability and consistency in this regard. Projects operate in schools, youth clubs, community centers and so forth. Incentives for the “educators” are sometimes included (e.g., testimonials, course credits or even money).

### 3.1. Sexual Health

A narrative review of peer education in sexual health [22] reported that projects were diverse in aims, objectives, methods, findings and levels of evaluation. This early review noted a lack of good evaluation and that knowledge gains were not the same as behavior gains. A more recent systematic review (predominantly of RCTs) [23] found 13 articles that met the inclusion criteria. Most interventions led to improvements in knowledge, attitudes and intentions. However, almost no overall benefit of peer education in behavior was found, and study quality was poor. Just one included study reported a reduced risk of chlamydia (ES 0.2). One study found some effects on girls but not on boys. 

Sexual health in developing countries was a matter of interest [24]. The often-simplistic model of social relations that underlay peer education interventions was questioned. This could have led to the reinforcement of gendered power relations and took no account of the social dynamics of poverty. In spite of the mismatch between rhetoric and reality, the appeal of peer education remained powerful, stemming from seeking to engage young people in a way that increased their autonomy and capacity. However, this paper was weak on actually reviewing the evidence. Pregnancy prevention and sexual health promotion for young people in Europe was reviewed [25], with a focus on peer education for HIV prevention, adolescent pregnancy prevention and promotion of sexual health. Just five adequate studies were identified, and few statistically significant changes found. There was no clear evidence of the effectiveness of peer education. No ESs were given.

A systematic review and meta-analysis of unprotected anal intercourse (UAI) among men who had sex with men [26] included 22 studies, including five RCTs, six quasi-experimental studies, six pre-and-post intervention studies and five cross-sectional intervention studies. Peer-led interventions reduced UAI (ES 0.27; *p* < 0.01). However, this was less so in RCTs and pre-post studies, and heterogeneity was large. Thus, peer-led HIV prevention interventions were effective, but efficacy varied by study design. This study in 2014 was the first to offer persuasive evidence for the effectiveness of peer education.

A review of Iranian adolescents’ educational needs for sexual and reproductive health [27] noted that Iran adolescents formed a large part of the population, so the issue had special importance. Thirty-seven articles were included, and adolescent sexual reproductive needs were categorized into three domains: adolescents (knowledge, attitude, information sources), school (curriculum content, time and manner of curriculum’s implementation, role of teachers) and family (role of parents and Islam domains). There were many unreliable sources of information and much incorrect learning, leading to a lack of or faulty awareness in adolescents, their parents and teachers. Because of shame, taboos and cultural and social beliefs, sexual reproductive health education for adolescents had been neglected in family and school. 

The effects of peer sexual health education on college campuses were reviewed [28], and out of 2503 hits, eight articles were included. Peer education was beneficial for increasing knowledge of sexual health topics. It also created some behavior change, such as increased condom use and HIV testing. Additionally, interventions specifically for women were more effective. A systematic review of school-based sexual health education for adolescents in Turkey [29] included six studies which met the inclusion criteria, but only one used the peer education method. However, all studies reported positive outcomes, although no study reported longitudinal outcomes. Negative attitudes towards sexual health education and hidden cultural resistance were the main barriers to the development of peer education.

Adolescent health programming in India, another country with a large youth population, was reported [30]. A rapid review of two existing government programs focused on four domains (governance, implementation, monitoring and linkages). Policy documents were scrutinized and interviews with officials conducted. The government programs had put adolescent health on the agenda for the first time, though inadequate human and financial resources limited impact. Services were not easily accessible to adolescents, and many were not even aware of them. Monitoring implementation quality was a challenge, as was training of helpers and managers. Further action needed to ensure that peer educators were properly trained, supported and retained. A systematic review of peer education programs for sexual and reproductive health in India was conducted [31]. Of over 1500 hits identified, only 13 were included in the review. Peer education was implemented, both as part of multi-component programs and as a stand-alone intervention. The outcomes were mixed, with both types having some statistically significant outcomes and others not. However, there were limited effects on behavior change.

### 3.2. Human Immunodeficiency Virus/Acquired Immune Deficiency Syndrome (HIV/AIDS)

An early review [32] examined literature on peer education in HIV/AIDS programs for youth. Peer involvement helped increase access to and acceptance of HIV/AIDS prevention messages. It also created the possibility of behavior change, but that was not guaranteed. Peer education for HIV prevention was reviewed in a national peer education program for HIV prevention in Vietnam [33]. Twenty (32%) of Vietnam’s provinces and urban areas had functioning peer education programs, and the coordinators of all 20 were interviewed. On-site reviews were also completed for 10 of the 20 programs, including interviews of peer educators and high-risk clients. Overall, 500 peer educators made 7000 contacts per month with high-risk persons, but many were contacted repeatedly. Despite this, some provinces with high numbers of HIV/AIDS clients had few peer educators, and few provinces targeted sex partners. The definition of peer education and composition of teams varied substantially; only one province included persons living with HIV/AIDS subjects as peer educators. Peer educators distributed information either through word of mouth, pamphlets or brochures, providing condoms and sometimes clean syringes and needles. Training was rarely provided.

A further review [34] focused on peer-led interventions to reduce HIV risk, synthesizing results from 24 evaluated peer-led programs in low- and middle-income countries. These programs showed success in effecting positive change in knowledge and condom use and some success in changing community attitudes and norms. Effects on other sexual behaviors and Sexually Transmitted Infection (STI) rates were mixed. Recommendations were given for successful community-based peer-led programs in low-income countries. A systematic review of adolescent behavior change interventions in sub-Saharan Africa [35] noted that although the area had just 12% of the world’s population, it had the highest burden of HIV: 70% of HIV infection and 80% of new infections among young people. Various intervention programs had been tried but with minimal translation into behavior change. Relevant studies were reduced to 17; three of these were RCTs and five quasi-experimental, while six were peer education programs. Eight interventions had positive outcomes in both knowledge and sexual practices, but other studies showed limited efficacy in behavior change. However, peer education seemed more effective than other psycho-social interventions in facilitating HIV risk reduction. 

A further review [36] focused on men who had sex with men (MSM) and trans-gender women in Southeast Asia. Only five of 575 screened studies met the inclusion criteria. Peer education was the most commonly employed intervention, although it was usually delivered as an element of a larger intervention package. There was a significant effect on at least one behavioral outcome measure (condom use, water-based lubricant use, number of sex partners, HIV prevention knowledge, willingness to use pre-exposure prophylaxis). A review of adolescent peer education programs for reducing HIV/STI Risk [37] noted that evidence for behavior change was mixed. Twenty-four quantitative and six qualitative studies were included. There was evidence of the effectiveness of adolescent peer-led HIV education programs on knowledge, attitudes, normative beliefs and self-efficacy, but studies were equivocal on sexual behavior. Nonetheless, peer educators and clients highly valued peer-led programs. 

A review of the effectiveness of peer education interventions for HIV prevention in developing countries [38] included 30 studies. Peer education interventions were significantly associated with increased HIV knowledge (ES 2.28), reduced equipment sharing among injection drug users (ES 0.37) and increased condom use (ES 1.92). However, they had a non-significant effect on STIs (but a high ES, 1.22). Peer education programs in developing countries were effective at improving behavioral outcomes. This was the first review to suggest comprehensive effectiveness of peer education in HIV/AIDS. The efficacy of HIV interventions among factory workers in low- and middle-income countries were investigated [39]. Thirteen articles were included, with two RCTs and 11 cohort studies. Peer education with community intervention increased the proportion of workers willing to take their partners to HIV counselling and testing. Policy intervention combined with peer education enhanced HIV knowledge, perceived condom accessibility and condom use with regular partners. The combination of multiple interventions achieved better efficacy than a single intervention. 

A systematic review and meta-analysis of peer education for HIV prevention among high-risk groups [40] included 60 articles with 96,484 subjects. Peer education was associated with a 36% decrease in rates of HIV infection among high-risk groups (ES 0.64). Peer education could improve rates of HIV testing (ES = 3.19) and condom use (ES = 2.66), while reducing equipment sharing (ES = 0.50) and unprotected sex (ES = 0.82). Importantly, peer education showed a consistent effect over 24 months on behavior change. This study showed that peer education had a long-term impact on behavior change among high-risk HIV groups. A review was reported [41] of the effectiveness of peer support for people living with HIV, including 20 RCTs comprising 7605 participants from nine different countries. Main outcomes showed better retention in care (ES 1.07, maintained at the 12-month follow-up), antiretroviral therapy (ART) adherence (ES = 1.06, maintained at three-month follow-up) and viral suppression (ES up to 6.24, maintained at six-month follow-up). The state of evidence for most other main outcomes (ART initiation, CD4 cell count, quality of life, mental health) was promising but too uncertain for firm conclusions. Overall, peer support was superior to routine clinic follow-up in improving outcomes for people living with HIV over the long term. 

### 3.3. Social Problems

#### 3.3.1. Drug Use

A systematic review of school-based drug prevention programs [9] included three meta-analyses, six studies examining mediating variables and 21 studies directly comparing prevention programs with or without specific characteristics. Interactive social influence methods had an ES of 0.16, considerably larger than that of non-interactive programs. There were larger effects for programs with a same-age peer as a leader, with 10 or fewer sessions, that were distributed over a longer period and that focused broadly on substance abuse. Combining these characteristics increased the ES to 0.72. An important mediator was a focus on a normative approach, including social prevalence knowledge, social acceptability knowledge, normative expectations and friends’ reactions to drug use. Peer-led interventions showed strong positive effects of booster sessions, but these resulted in worse outcomes for teacher-led interventions. Overall, peer-led programs were more effective than adult-led programs (ES = 0.24).

A systematic review of peer-delivered support services for people in recovery from alcohol and drug addiction [10] had 1240 hits, but only nine studies were included. Programs were in various settings, including peer-run drop-in centers, peer-run recovery community organizations and medical outpatient clinics. Most studies reported statistically significant findings, indicating behavioral change in participants in substance use, a range of recovery outcomes or both. Additional research was needed on the effectiveness of different approaches and types of peer support services with regard to the amount, intensity, skill level of the peer, service context and effectiveness among different target populations.

#### 3.3.2. Obesity

A systematic review investigated the impact of school-based, peer-led nutrition education initiatives [14] in 17 articles (relating to 11 programs) in Canada (24%) and the United States (76%). Outcome measures included healthy eating knowledge (*n* = 5), self-efficacy or attitudes towards healthy eating (*n* = 13), dietary measures (*n* = 9) and body mass index (BMI) (*n* = 4), all of which tended to improve. Four studies showed that peer education programs were well received by students, staff and parents. Two studies reported increased self-esteem. One study reported improved body image but no differences from the control group. Thirteen studies used diet or health behavior change as an outcome measure, and 11 found improvement in this immediately post-program. Changes included increased fruit/vegetable intake, reduced sugar-sweetened beverage intake, reduced fat intake and improved self-reported habit and behavior scores. However, these changes tended not to be maintained in the longer term (four studies). Results were promising in the four studies that investigated anthropometric measures, showing either decreases in BMI percentiles, changes in BMI related to normal growth or increases that were less than in the control group. Programs were generally well received, but the long-term maintenance of positive impacts was a challenge. 

A further meta-analysis [12] reported on effects of peer support with individuals that were overweight and with BMI, waist circumference, blood pressure, quality of life, social support and depressive symptoms. A significant improvement in weight was found, which was more than for those receiving usual care (ES 0.78, *p* = 0.02). Peer support was associated with a significant decrease in BMI (ES 0.16, *p* = 0.04). However, there was no statistically significant improvement in waist circumference, systolic blood pressure, diastolic blood pressure, quality of life, social support and depressive symptoms. A systematic review on peer-supported lifestyle interventions on body weight, energy intake and physical activity in adults [13] found 2435 hits, of which 65 articles were included (participants = 15,673). Peer intervention resulted in a significant reduction in weight (ES 1.05, 28 studies), BMI (ES 0.24, 25 studies) and waist circumference (ES 0.75, 12 studies) and a significant increase in physical activity (ES 0.20, 41 studies). 

#### 3.3.3. Smoking 

A review of RCTs on school-based smoking prevention in adolescents in developing countries [8] noted that in such places, around 90% of smokers started consuming tobacco before 18 years. Smoking in the traditional way was investigated along with cigars, smokeless tobacco, and hookah or shisha. The search found 594 hits, but only seven were included, and only three of these were about peer education. Peer intervention could change behavior while increasing knowledge. Peer education process evaluations reported participants finding peer-led sessions more enjoyable, feeling peers were credible sources of information and preferring peer-led sessions. Comparing classical teaching methods with peer education, the average post-test score of students with peer education increased significantly. The possibility of smoking was lower in the group receiving peer education interventions as compared to the control group.

#### 3.3.4. Alcohol Abuse

A systematic review and meta-analysis on peer-led interventions to prevent tobacco, alcohol and/or drug use among young people aged 11–21 years [11] included only RCTs. Seventeen eligible studies were found, half of which were school-based studies targeting tobacco use among adolescents, representing 13,706 young people in 220 schools. The odds of smoking were lower among those receiving the peer-led intervention compared with controls (ES = 0.78, *p* = 0.040). Pooling of six studies representing 1699 individuals in 66 schools showed that peer-led interventions were associated with benefit in relation to alcohol use (ES = 0.80, *p* = 0.036), while three studies (*n* = 976 students in 38 schools) linked peer education to lower odds of cannabis use (ES = 0.70, *p* = 0.034). However, the evidence base was limited, with many small studies of low quality. Nonetheless, peer education could be effective in preventing tobacco, alcohol and cannabis use among adolescents. 

### 3.4. Medical Conditions

A systematic review of RCTs of peer education among adults with type 2 diabetes [42] included seven studies with reasonably high quality ratings. There was no consistent design, setting or outcome measurement. Two types of intervention were compared to traditional diabetes education: face-to-face or a combination of face-to-face and telephone/texting. The most common outcome measure was HbA1c (average blood glucose levels). Two of six studies showed statistically significant improvement in HbA1c between intervention and control groups. A statistically significant increase in diabetes knowledge was found in two of five studies. The results were mixed, but clearly, peer education could be successful in behavior change. Professional education was not superior to peer education. 

Peer education programs for adolescents with asthma were examined [43], and 16 databases searched for RCTs with participants 10–19 years old with asthma. Of the 1887 articles retrieved, just four (*n* = 1937 participants) met the inclusion criteria. There was a statistically insignificant increase in participants’ quality of life, but the ES was quite large (0.70). There was also a small, insignificant change in lung function, but again, the ES was large (ES = 1.36). Thus, peer education programs could improve quality of life or lung function for adolescents with asthma. 

Peers with spinal cord injury (SCI) were the subject of a scoping review [44]. Health implications could arise from direct nerve damage, secondary conditions and the increased probability of a sedentary lifestyle. Eight studies were included and described in three themes: timing and focus, role of peer educators and outcomes. Half of the studies included peer education, but all reported positive outcomes. Peer education seemed promising in assisting people with SCI and raising their self-efficacy. A more general systematic review of peer education on self-care behaviors of patients [45] found 39 articles, and 10 were included. With patients with diabetes, peer education resulted in increased self-care, better use of medication and reductions in the need for insulin. Peer education also seemed effective in reducing anxiety in patients undergoing coronary artery bypass surgery. 

A scoping review of peer-led physical activity (PA) interventions with young people [46] found 2237 hits, of which 651 were duplicates. Measures such as BMI were excluded, as were samples smaller than 50. Finally, 43 studies were included, of which 18 investigated intervention effects. Five of these investigated effects on sedentary behavior, six self-efficacy and two mental wellbeing. Six studies were RCTs, but only two featured any kind of follow-up. Eight studies reported an intervention effect on PA measured objectively with devices such as accelerometers and pedometers. Four studies reported intervention effects on fitness and object control skills measured with physical tests. Seven studies used self-report questionnaires. Only two studies reported insignificant intervention effects on fitness. Thus, in the majority of cases, peer leadership could increase physical activity for youth and children.

### 3.5. Prisons

A systematic review of peer education to promote health and healthy behavior in prisons [47] located 3033 hits, leading to 46 articles, of which 10 were included. Peer education in prisons could impact attitudes, knowledge and behavioral intention regarding HIV risk behavior. Findings were inconclusive for illicit drug use and injecting practice. There was little research on peer education impacting mental health, obesity, diet, smoking or the self-management of chronic physical diseases in prison. Likewise, a systematic review of general peer education for healthy behavior [48] noted that prisoners experienced significantly worse health than the general population. Nineteen databases were searched and both qualitative and quantitative syntheses conducted. Fifty-seven studies were included, but many were of poor methodological quality. Peer education appeared effective at reducing risky behaviors and was acceptable to recipients. Being a peer deliverer was consistently associated with positive effects. Peer educators were as effective as professional educators in HIV prevention. Peer delivery was preferred to professional delivery by recipients. Peer deliverers demonstrated empathy having shared lived experiences, were non-judgmental, were trusted by prisoners and offered more time than staff. Prisoners felt more at ease talking to fellow prisoners and found them more accessible. 

HIV risk and prevalence in U.S. prisons was the focus of a systematic analysis [49]. The HIV risk in prions was five times that of the general population. A total of 27 articles met the inclusion criteria; 12 of these dealt with peer education. The interventions showed positive effects on a variety of key outcomes, including HIV knowledge, intention to change risky behavior, perceived risk of infection and HIV test rates. Interactive education programs were more effective than passive programs. Peer education was particularly effective at reducing HIV risk behaviors and had significant benefits for peer educators. One study of women inmates suggested that communication skills were just as important as knowledge of HIV and safe sex. 

### 3.6. General

An early review of peer education on college campuses was conducted [50], although at that time, few evaluation studies had been done. Another review examined school-based programs and compared them to peer-led and adult-led initiatives [51]. Thirteen studies were included, mostly of smoking, but other problems were featured. Seven studies reported gains in knowledge and attitudes. Peers were more effective in altering attitudes than professionals. Eleven studies of behavioral change reported that peer-led interventions were more effective than adult-led, and only one found the opposite. Studies were of poor methodological quality. A subsequent narrative review [52] noted that peer programs were widely used for health education content, but evaluations were few. Green [53] likewise reported some theories but only a few studies. 

The first review with substantial content was that of Harden et al. [54]. This was a systematic review of both quantitative studies of effects and qualitative studies of process. Four hundred and thirty hits were identified, of which 64 (49 outcome and 15 process) met the inclusion criteria. Four targeted young people aged over 16 and eight young people under 16. Two interventions were in a community setting, and the other 10 were in educational settings. Four interventions focused on sexual health, five on the prevention of smoking, one on asthma education, one on violence prevention and one on the prevention of testicular cancer. Seven of the interventions were effective for at least one behavioral outcome. Three were effective for non-behavioral outcomes (knowledge, belief or attitudes), and two were unclear. Only 12 (24%) were deemed methodologically sound. However, no ESs were given. The majority of process evaluations examined implementation (*n* = 9, 60%) and acceptability (*n* = 10, 67%). Thus, the evidence for the effectiveness of peer-delivered health promotion for young people was mixed. More systematic research was needed. 

A review of peer education for adults [55] investigated 25 RCTs. Studies were grouped by outcome measures, with ESs ranging from −0.50 to 2.86. Peer-based interventions facilitated important changes in behavior, including physical activity, smoking and condom use, with small- to medium-sized ESs. However, the evidence was mixed, and there was heterogeneity in the methods and doses between studies. A narrative review of peer education for adolescents [56] noted that there were some successful programs, but the evidence was mixed. No ESs were given. Designing such programs required planning, training, supervision and evaluation. Likewise, a narrative review of peer education in adolescents [57] included 53 articles. Factors in peer education were characteristics of the peer educators, including personal skills and communication; characteristics of the educational program, including theoretical foundations, program transparency, program sustainability, adolescents’ comprehensive participation, and evaluation and monitoring; and structural characteristics, including supportive structures and financial structures. This study featured a PRISMA chart but offered no ESs and indeed no evidence of effectiveness. 

A systematic review [58] examined 116 RCTs and described study design, participants, type of intervention(s), peer role(s), outcomes assessed, measures used and effects extracted. There were more null than positive effects, with notable exceptions. Group-based interventions using peers as educators or group facilitators commonly improved knowledge, attitudes, beliefs, perceptions, social health, connectedness and engagement. Dyadic peer support influenced behavior change. A review with a similar focus but completely different methodology [59] discussed Youth Health Champions (YHC) who used a peer education approach for promoting health and wellbeing in youth settings. The European YHC project intended to make peer education training more inclusive and accessible. A literature review was undertaken prior to launch, including a number of systematic reviews, project reports and training manuals, together with 15 case studies of peer education in 11 European countries. Peer education was widely used for health promotion with young people. There was no consensus on the impact of peer education on behavior change, but there was strong evidence of an increase in healthier lifestyles and dissemination of information on sexual health, HIV prevention and domestic violence. 

A scoping review on peer-led health promotion programs for adults was reported [60], including 55 studies. Most (*n* = 32) used qualitative research. Health problems addressed were mostly related to chronic diseases (*n* = 19) and HIV and STIs (*n* = 13). Other topics included nutrition (*n* = 5), safe sexual behaviors (*n* = 4), safe drug injection (*n* = 4), healthy lifestyles (*n* = 3), smoking cessation (*n* = 3), body image and weight management (*n* = 2), mental health (*n* = 2), prevention of vision impairments (*n* = 1), injury prevention (*n* = 1), end of life care (*n* = 1), and physical activity (*n* = 1). Most papers reported on participant responsiveness (i.e., participation rates, satisfaction or engagement, *n* = 25), peer leaders’ responsiveness (*n* = 23) and program fidelity (*n* = 18). Other papers evaluated implementation more generally (*n* = 28). This study gave an elaborate framework for coding the studies on various parameters, but the question of effectiveness was not really addressed. 

Peer-led health programs for indigenous youth were systematically reviewed [61] and 24 included studies reported 20 interventions involving young indigenous people delivering health information to age-matched peers. There was only one RCT, the majority being pre–post studies. There was evidence of changes in behavior, knowledge or attitude. Changes in behavior included increased STI testing, increased use of health services and decreased alcohol and/or other substance use. Effects on knowledge included increased awareness of sexual health issues, improved healthy lifestyle knowledge, better understanding of the dangers of drug abuse and/or addiction and better understanding of mental health issues and how to support someone feeling depressed. Attitudinal changes included improved self-confidence, self-esteem and/or self-perception; increased intention to reduce/abstain from substance use and increased intention to use condoms. There were methodological limitations in a majority of studies. 

Peer education with Iranian adolescents [62] was examined in 20 articles (*n* = 6652 adolescents) meeting the inclusion criteria, in which the effect of peer education was reviewed systemically in four categories: prevention of diseases, mental health, nutritional behaviors and high-risk behaviors. In all categories, there was an equal or greater effect of peer education on knowledge, attitude, practice, self-efficacy and health behavior of adolescents, compared to education by teachers, health personnel, lectures, pamphlets and booklets. Only the effect of education by a doctor was greater than that of peer education.

Peer-facilitated community-based interventions for adolescent health in low- and middle-income countries were investigated [63], noting that adolescents aged 10–19 represented one-sixth of the world’s population and had high morbidity, particularly in low-resource settings. Twenty RCTs involving 61,014 adolescents featuring peer education and peer counselling by young people aged 10–24 were included. Outcomes included infectious and vaccine-preventable diseases, under-nutrition, HIV/AIDS, sexual and reproductive health, unintentional injuries, violence, physical disorders, mental disorders and substance use. Fourteen studies tested interventions linked to schools or colleges. Four studies had HIV-related outcomes, but none reported reductions in HIV incidence. Nine studies had clinical sexual and reproductive health outcomes, but only one reported a positive effect. Three studies had violence-related outcomes, two of which focused on reductions in physical violence by school staff and by adolescents. Seven studies had mental health outcomes, four of which reported reductions in depressive symptoms. Finally, there were eight studies on substance use, four of which reported reductions in alcohol consumption and smoking or tobacco use. Thus, 11 of 31 double-counted studies reported positive outcomes, so results were mixed.

## 4. Results: Peer Counselling and Peer Support

Talking your problems through with a friend is hardly a new concept, but it is increasingly being seen as a preferred means of support. However, it is not an option if your problem is that you do not have a friend. Peer counselling has indeed been around for a long time. In 1974, the nascent field was reviewed [64]. More programs began to appear in the 1980s, such as TIP, the Total Involvement Program for Peer Facilitators [65]. Bond [66] reported on “Knowing You–Knowing Me”, involving self-development goals for both the counselled and counsellors. There was increasing interest in developing peer counselling facilities in youth and community centers and other neighborhood drop-in facilities, and the development of peer counselling into a “city-wide” program was reported [67]. Peer counselling programs are built on spontaneous, naturalistic peer support and seek to equalize access opportunities and deepen and widen the impact.

However, several cautionary notes were sounded [4], reminding us that you cannot counsel someone unless they want to be counselled. Managing the public image of the peer counselling program is important, so any stigma is avoided. Also peer counselling should not be an agent of social control; it should empower the recipient to fulfil their own goals. Peers are less likely than professionals to be judgmental and pathologize the behavior of clients, particularly when they are members of an ethnic minority, and perhaps the peer educator could also be from that ethnic minority. Additionally, being a peer counsellor was valuable as a vehicle for learning about yourself. As with peer education, peer counselling is of course not confined to children and young people; those of any age can participate, and there is a significant literature on the training and deployment of senior citizens as peer counsellors, to the benefit of both parties. 

### 4.1. Mental Health

A relatively early review of peer support within mental health services [6] noted that seven RCTs with 301 participants used varied outcome measures and had mixed findings. The wider evidence base showed some impact on social functioning, social support and community integration, self-esteem and confidence, stigmatization, empowerment and quality of life, symptom distress and hospital admission rates and re-admission rates. There were also benefits for the peer helpers, especially in terms of raised self-esteem. Training, supervision, support and monitoring for the peer supporters were all necessary. There were some follow-up studies. Another review [5] focused particularly on depression. In a meta-analysis, seven RCTs of peer support versus usual care for depression involving 869 participants were identified. Peer support interventions were better at reducing depressive symptoms, with an ES of 0.59 (*p* = 0.002). Seven RCTs compared peer support to group cognitive behavioral therapy: there was no significant difference, but an ES of 0.10 indicated a small effect for peer support. Peer support interventions did reduce symptoms of depression.

Online peer-to-peer suicide prevention programs for young adults were reviewed [68], noting that worldwide suicide was the second most prevalent cause of death among 15–25-year-olds. Thirteen studies of peer counselling meeting inclusion criteria generally showed positive results, concerning implementation, satisfaction and efficacy. However, many studies had methodological deficiencies. Another review [7] noted that earlier reviews incorporated different modalities of peer support (e.g., group and one-to-one), had substantial heterogeneity and quality issues and offered inconsistent evidence of effectiveness. This study focused only on one-to-one peer support for adults using mental health services. Twenty-three studies reporting 19 trials were eligible (*n* = 3329 participants). The risk of being hospitalized was reduced by 14% for those receiving peer support (RR = 0.86). The length of stay in hospital was not significant. Recovery, empowerment and satisfaction with services all had modest positive RRs (0.19–0.23). There was no impact on clinical symptoms or service use. 

### 4.2. Breast-Feeding

A systematic review [69] analyzed RCTs assessing the effectiveness of peer counselling for breastfeeding in improving rates of breastfeeding initiation, duration, exclusivity and maternal and child health outcomes. Twenty-six publications were included. The overwhelming majority of evidence from RCTs indicated that peer counsellors effectively improved rates of breastfeeding initiation, duration and exclusivity. Peer counselling interventions also significantly decreased infant diarrhea and significantly increased the duration of lactational amenorrhea. Thus, breastfeeding peer counselling initiatives were effective. A review of peer counselling regarding breast feeding in Uganda was reported [70], noting that there were limited data on such efforts in sub-Saharan Africa. A breastfeeding intervention targeted mothers and their 0–6-month-old children. In both control and intervention groups, women could access standard health facility breastfeeding promotion services. However, peer counselling more than doubled mother-reported breastfeeding prevalence, but there was no observable impact on diarrhea prevalence. Since the intervention significantly increased the prevalence of breastfeeding, it could be adopted if benefits other than reducing diarrhea were important.

A subsequent systematic review and meta-analysis of interventions to promote exclusive breastfeeding among young mothers was reported [71]. In high-income countries, younger mothers were less likely than older mothers to exclusively breastfeed or to exclusively breastfeed for a long duration. Of 955 hits, only nine studies met the inclusion criteria. Seven studies were RCTs, and two were quasi-experimental. Eight were from the United States. Most studies included a combination of strategies. Follow-up time varied from three weeks to six months. Only three of eight studies reported an effect on exclusive breast feeding. High rates of attrition and formula supplementation among the participants made it difficult to detect a statistically significant effect.

### 4.3. Medical Conditions

Peer support interventions for breast cancer patients were systematically reviewed [72]. Given the heterogeneity of such interventions, this study aimed to categorize, assess, and synthesize existing evidence from RCTs to clarify effects of different types of peer support on breast cancer patients. Of 1494 studies screened, 15 met the criteria for inclusion, involving 1695 breast cancer patients. Overall, there were more positive effects than invalid or negative effects. However, unstructured/unmoderated group peer support interventions and internet-based models without peer training had nil or a negative effect. Nonetheless, there were promising positive effects on stress management, quality of life and healthy behaviors. This study was followed [73] by a systematic review and meta-analysis of peer counselling to compensate for racial/ethnic disparities in colorectal cancer screening. Low implementation of such screening in ethnic minorities was the main reason for disparities in morbidity and mortality. Thirteen studies (*n* = 8090 participants) met the eligibility criteria. Peer support could increase colorectal cancer screening implementation and raise awareness and intention to undergo the screening in ethnic minorities more significantly than fecal occult blood test outreach, print, and usual care. Peer support achieved the best results with Asian Americans, while church-based programs required improved management and fidelity.

## 5. Discussion 

### 5.1. Summary

Table 1 gives a summary of the overall picture. The quality of studies was highest for the peer education programs in HIV/AIDS and Social Problems and weakest in the peer education General category.

#### 5.1.1. Peer Education

The picture on evaluation in sexual health is that early reviews lamented the lack of evidence or reported that the evidence was not positive, the intermediate reviews lamented the lack of impact on behavior despite the evidence of knowledge gains and the latest broad-spectrum reviews provide better evidence on effects in terms of both knowledge gains and behavioral effects. Peer education might be more effective with women than men. In developing countries, the picture is less positive, the results more mixed and cultural attitudes can be a major problem, although Turkey has shown excellent results.

Sixty percent of the review literature on peer education for HIV/AIDs amelioration and prevention stems from developing countries, where the problem in relation to population size is much greater, and resources to address it are less available. Nonetheless, in developing countries, there is evidence not only of knowledge gain, but also of behavioral change, in at least some of the target behaviors (including condom use, reduced equipment sharing and number of sex partners). However, effects on other behaviors were less certain (e.g., acquisition of STIs). Early broad-spectrum reviews were equivocal on behavior change, but the most recent studies (e.g., [41,73]) showed that peer education led to improved rates of HIV testing, improved condom use, reduced unprotected sex, reduced equipment sharing, decreased rates of HIV infection, improved ART adherence, improved viral suppression and improved retention in care, with effects lasting from 3–24 months.

Turning to peer education with social problems, in regard to drug abuse, peer-led sessions were effective and more effective than professional-led sessions. Participants showed improvements in substance use and a range of recovery outcomes. Regarding obesity, healthy eating knowledge, attitudes towards healthy eating, intentions, dietary measures, waist circumference, weight and body mass index (BMI) all improved. Programs were well received by students, staff and parents. However, these changes tended not to be maintained in the longer-term (three months to two years). Programs targeting physical activity reported a significant improvement. Regarding smoking, a review of school-based smoking prevention in adolescents in developing countries showed increased knowledge and changed behavior. Peer-led sessions were more enjoyable, more credible and were preferred. Peer education was more effective than professional education. Regarding alcohol abuse, the odds of abuse were lower among those receiving the peer-led intervention compared with controls. Additionally, three of these studies also suggested an impact on cannabis use.

Considering peer education with various medical conditions, in adults with type 2 diabetes a minority of studies gave positive effects. Peer education could improve lung function and quality of life for patients with asthma. Peer education with spinal cord injury victims showed a high degree of success. A more general review of peer education had mixed results but good effectiveness with diabetes and cardiac patients. Peer-led physical activity interventions involving young people demonstrated mostly positive outcomes.

Peer education in prisons could impact on attitudes, knowledge, behavioral intention regarding HIV, reduced risky behavior and HIV test rates. Peer education was preferred to professional delivery and was as effective as professional education. Being a peer educator was consistently associated with positive effects. However, findings were inconclusive for the impact on illicit drug use and injecting practice.

Turning to the “General” category, early reviews were weak, with only one study [51] reporting any behavioral change. Even in later years, three reviews offered virtually no evidence on knowledge or behavior change. Three even later studies had mixed results, with a minority of studies showing effects for behavioral change, although effects for knowledge and attitudes were more common. Finally, the most recent three reviews were more positive, reporting not only knowledge changes, but also widespread behavior changes, in, for example, physical activity, smoking and condom use. However, in this section, the positivity of a review did not relate directly to the age of the review, as was the case with other sections; perhaps generality led to an excess of heterogeneity.

#### 5.1.2. Peer Counselling and Support

In the area of mental health, an early review on the effect of peer counselling and/or support on general mental health had mixed results, but a later one showed that the hospitalization rate reduced and that recovery improved. Other studies showed that peer counselling reduced depression and improved suicide prevention. In relation to breast-feeding, in general, effects were positive in increasing breast-feeding and reducing diarrhea (although diarrhea did not reduce in Uganda). High-income younger mothers showed some effects on exclusive breast-feeding, but many supplemented with formula. In terms of medical conditions, peer counselling and/or support was effective for most breast cancer patients. In ethnic minorities, peer support could raise awareness and intention to undergo colorectal cancer screening and increase actual screening implementation.

### 5.2. Strengths and Limitations

This search was restricted to a modest number of databases. However, it was supplemented with manual searches, which were necessary given the confused nomenclature which surrounds this topic. Extensive searching was required to find reviews on smoking, alcohol abuse, drug-taking and obesity. I noted that there was relatively little evidence of concern with regards to implementation integrity or fidelity, although a number of studies noted marked heterogeneity between studies. Furthermore, there was little evidence on the sustainability of programs over time. Where there was follow-up, it was usually for three to six months, with longer follow-up lacking. Additionally, most of the reviews followed a narrow medical model of identifying a problem and then seeking to fix it. In other words, the programs reported tended to be reactive to specific problem issues, and there was very little on more positive pro-active or preventive measures regarding health and well-being. The studies on developing physical activity were an exception to this [13,14,45].

The quality of reviews was very various, with a tendency for later reviews to be better, but it was by no means an even one. The HIV/AIDS section was particularly strong (see [34,35,38,40,41]), with later reviews tending to be stronger. Obesity reviews were also strong, and again, later reviews were stronger [12,13]. Mental Health reviews were also strong [5,7], and again, later reviews tended to be stronger. However, in other areas, there were fewer reviews which could be considered strong, irrespective of the number of reviews in their area, and these bore little relationship to the date of publication (see [54] in General, ref. [9] in drug abuse, ref. [69] in breast-feeding, ref. [26] in sexual health, ref. [11] in alcohol abuse and [43] in medical conditions: asthma). Some areas had no studies which were considered strong (Smoking and Prisons).

### 5.3. Implications for Action

Peer education and peer counselling and support should be further extended and more carefully researched in a number of areas. In fact, there are some promising results in every area reviewed above, so there should be no exceptions to this. However, there are implications for the quality of future research. Narrative reviews are no longer needed. Systematic reviews and meta-analyses are replicable and more reliable. Even in these, the quality of searches is highly variable, not least in the choice of keywords, databases and quality criteria, and researchers intending such a venture would do well to study the methodology of previous reviews, which this paper facilitates. Additionally, meta-analyses in particular involve the exclusion of a great many studies and consequent risks of introducing unintentional bias. Work with special populations not only includes at-risk groups in under-developed countries, but also prisons, where the context is radically different, and other special contexts may be identified. There were many studies reporting that peer education was more effective than professional-led education and preferred by clients. Of course, the professionals might now be re-tasked to train, supervise and monitor peer-led projects, but the quality of this professional action should certainly be the subject of research.

Regarding the action implications for practitioners, it is clear that the most positive studies review papers which give implications for the organization of peer education and counselling. These should be studied before embarking on any project in this area. The quality of program structure, management, initial training, supervision, support, monitoring and retention are all factors which require not only planning, but also resourcing. Equally, it is important to consider cost-effectiveness; peer education and counselling are certainly not free when all the costs are considered.

Turning to political implications, it will be important that bodies considering funding initiatives carefully consider the research literature as well as the detailed plan for the implantation of the project before agreeing to fund it.

Thus, in all cases, the quality of program structure, management, training, supervision, support, monitoring and retention is important, and more attention should be given to these factors. Monitoring of implementation fidelity or integrity is important and should be reported. Longer-term follow-up (up to two years) should be carried out much more frequently and reported.

## 6. Conclusions

Peer education and peer counselling are now showing good evidence of effectiveness but only in relation to certain areas of human activity. This needs to be generalized to more areas of activity. They are certainly feasible but difficult to manage and even more difficult to quality control, given that much activity happens outside of easily observable contexts. Serious and detailed thought should be given to organizational factors before starting any new venture. In terms of effectiveness, more recent reviews have tended to find changes in both knowledge and behavior, so in principle, the method is effective. Of course, that does not mean it will be effective right there where you are, so careful process and outcome evaluation will be necessary to ensure you stay on the right track. There is certainly considerable future potential, but peer education and counselling are not to be taken lightly. If research is to continue to show increases in knowledge and behavior, most new projects will need to be much more carefully organized.

## Figures and Tables

**Table 1:** Categorization of the Review Studies by Type of Problem.

Type of Problem	Total Number of Reviews	Number of Types of Review: Narrative, Systematic or Meta-Analysis *	Number of Studies with Effect Sizes or Equivalent	Effect Sizes or Equivalent Mean in ()
PEER EDUCATON				
Sexual Health	10	Narrative 5 Systematic 5 Meta-analysis 1	1	0.27
HIV/AIDS	10	Narrative 4 Systematic 4 Meta-analysis 3	3	2.28, 0.37, 1.92, 1.22 3.19, 2.66, 0.50, 0.82 1.07, 1.06, 6.24 (mean = 1.94)
Social Problems	7	Narrative Systematic 5 Meta-analysis 3	4	0.16, 0.72, 0.24 0.78, 0.12 1.05, 0.24, 0.75, 0.20 0.78, 0.80, 0.70 (mean = 0.55)
Medical Conditions	5	Narrative 1 Systematic 3 Meta-analysis 1	1	0.70, 1.36 (mean = 1.03)
Prisons	3	Narrative Systematic 3 Meta-analysis	0	
General	14	Narrative 8 Systematic 5 Meta-analysis 1	1	−0.50, 2.86 (mean = 1.18)
PEER COUNSELING				
Mental Health	4	Narrative 1 Systematic 2 Meta-analysis 1	2	0.59, 0.10 0.86, 0.19, 0.23 (mean = 0.39)
Breast-Feeding	3	Narrative 1 Systematic 2 Meta-analysis1	0	
Medical Conditions	2	Narrative Systematic 2 Meta-analysis1	0	

Peer support reviews were merged with the peer counselling reviews. * Some studies included more than one type of review, so numbers may not match.

## Data Availability

The reviews in this paper may all be obtained from the author on request.
